# Letter from the Editor in Chief

**DOI:** 10.19102/icrm.2024.15076

**Published:** 2024-07-15

**Authors:** Devi Nair


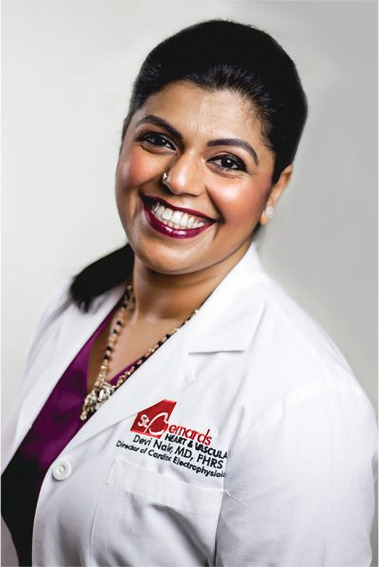


Dear readers,

I am honored and excited to step into the role of Editor-in-Chief for *The Journal of Innovations in Cardiac Rhythm Management*, beginning this July 2024. It is a privilege to follow in the footsteps of Dr. Moussa Mansour, whose remarkable leadership over the past decade has enabled the journal to achieve significant milestones, such as indexing in Scopus/Embase in 2019 and PubMed in 2020 and a 60% increase in original research submissions to date. His dedication and commitment have set a high standard, and I am eager to continue building upon this strong foundation.

As we move forward, I want to assure our readers and contributors that the journal’s mission remains steadfast: to provide the most current, high-quality research and educational content in the field of cardiac rhythm management. Our focus will remain on covering both the practical and innovative aspects of clinical cardiac electrophysiology, ensuring that the information we provide is not only cutting-edge but also applicable in clinical practice.

I am particularly excited about the advancements and ongoing research in pulsed-field ablation (PFA) for atrial fibrillation (AF), as highlighted by Dr. Mansour in his final letter in June 2024.^1^ The studies presented at the Heart Rhythm Society’s annual scientific meeting, such as the Assessment of Safety and Effectiveness in Treatment Management of Atrial Fibrillation with the Biosense Webster IRE Ablation System (admIRE) trial and the Treatment of Persistent Atrial Fibrillation with Sphere-9 Catheter and Affera Mapping and Ablation System (SPHERE Per-AF) study, underscore the significant impact of PFA on the practice of AF ablation. These findings not only validate the efficacy and safety of PFA but also promise to accelerate its adoption as a superior energy source for AF ablation.

Looking ahead, we aim to expand the journal’s reach and impact by continuing to publish groundbreaking studies and fostering an environment of rigorous scientific discourse. I encourage researchers and clinicians to submit their work and share their findings with our global readership.

I would like to express my gratitude to Dr. Mansour for his exceptional leadership and to the entire editorial team for their unwavering dedication and support. I am confident that, together, we will continue to advance the field of cardiac rhythm management and uphold the journal’s reputation for excellence.

Thank you for your continued support and commitment to *The Journal of Innovations in Cardiac Rhythm Management*. I look forward to embarking on this new journey with you and to the exciting developments ahead.

Best regards,


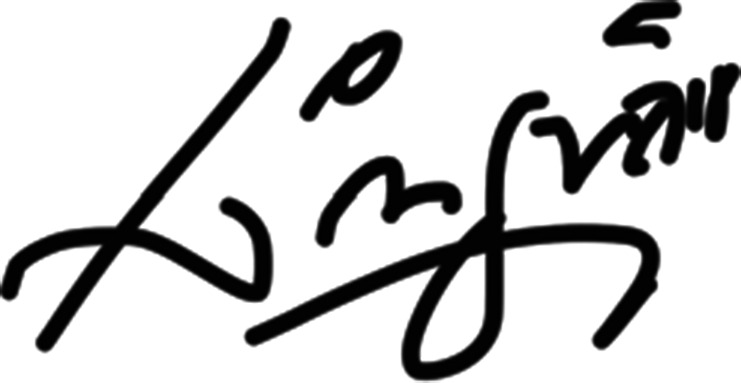


Dr. Devi Nair, md, facc, fhrs

Editor-in-Chief

*The Journal of Innovations in Cardiac Rhythm Management*

Director of the Cardiac Electrophysiology & Research,

St. Bernard’s Heart & Vascular Center, Jonesboro, AR, USA

White River Medical Center, Batesville, AR, USA

President/CEO, Arrhythmia Research Group

Clinical Adjunct Professor, University of Arkansas for Medical Sciences

Governor, Arkansas Chapter of American College of Cardiology

drdgnair@gmail.com
